# Why Not Enliven the Annual Meeting?

**Published:** 1906-03-03

**Authors:** 


### WHY NOT ENLIVEN THE ANNUAL MEETING?

We have often been struck, in reading the reports of proceedings at the annual meetings of various hospitals, that habit so dominates the minds of most of the men immediately responsible for the conduct of the committee's business, that they fail to make these occasions as interesting and profitable to the institutions as they should in fact prove. This habit creates a definite and circumscribed view, which makes the speakers strive to so handle the facts of the year's work as to give prominence to the points which in our judgment those facts do not always justify. This is very apparent in regard to the efforts invariably made to show that, whatever the receipts may have been, they are in fact inadequate, though they may, and sometimes do, exceed the annual expenditure for the particular year. We believe this to be a grievous mistake from the hospital's point of view, because nowadays the majority at any rate of the larger givers prefer to support those institutions the managers of which show a determined resolve to make the accounts of each year balance. In other words, public feeling now demands that excessive deficits, and indeed any deficit, shall as far as possible be avoided by sound management and foresight.

The public dinner habit, though it continues in this country, has lost much of its freshness, and we believe that if the breath of life could be given to the dead bones of the ordinary annual meeting of a hospital, it might be made in the present day more effective as an agency for raising a definite sum of money, and permanently benefiting the finances of the charity, than the festival dinner. Some years ago we made some successful experiments which fully confirmed the accuracy of this view, and we could wish that the managers of every hospital would give the question the careful consideration which it undoubtedly merits. With the object of furthering this movement we have ventured to precede the notice of each annual meeting of some of the principal hospitals with a few critical notes, so as to illustrate the point and drive it home. One thing is certain, and that is that every committee which is wise will make it their business to secure one or two men who are not engaged in the management and who will bring to bear fresh minds upon the condition and needs of their hospital, to which they will give expression in speech at the annual meetings. In this way the monotony arising from the habit into which the managers themselves unconsciously and possibly necessarily fall may at any rate be obviated, and the welfare of the hospital greatly promoted.

